# Signatures of adaptive decreased virulence of deformed wing virus in an isolated population of wild honeybees (*Apis mellifera*)

**DOI:** 10.1098/rspb.2023.1965

**Published:** 2023-10-25

**Authors:** Allyson M. Ray, Emma C. Gordon, Thomas D. Seeley, Jason L. Rasgon, Christina M. Grozinger

**Affiliations:** ^1^ Department of Entomology, The Pennsylvania State University, University Park, PA 16802-1503, USA; ^2^ Department of Biological Sciences, Vanderbilt University, Nashville, TN 37240-0002, USA; ^3^ Department of Neurobiology and Behavior, Cornell University, Ithaca, NY 14850, USA

**Keywords:** virulence, deformed wing virus, *Apis mellifera*, *Varroa destructor*, virus evolution, mite-surviving honeybees

## Abstract

Understanding the ecological and evolutionary processes that drive host–pathogen interactions is critical for combating epidemics and conserving species. The *Varroa destructor* mite and deformed wing virus (DWV) are two synergistic threats to Western honeybee (*Apis mellifera*) populations across the globe. Distinct honeybee populations have been found to self-sustain despite *Varroa* infestations, including colonies within the Arnot Forest outside Ithaca, NY, USA. We hypothesized that in these bee populations, DWV has been selected to produce an avirulent infection phenotype, allowing for the persistence of both host and disease-causing agents. To investigate this, we assessed the titre of viruses in bees from the Arnot Forest and managed apiaries, and assessed genomic variation and virulence differences between DWV isolates. Across groups, we found viral abundance was similar, but DWV genotypes were distinct. We also found that infections with isolates from the Arnot Forest resulted in higher survival and lower rates of symptomatic deformed wings, compared to analogous isolates from managed colonies, providing preliminary evidence to support the hypothesis of adaptive decreased viral virulence. Overall, this multi-level investigation of virus genotype and phenotype indicates that host ecological context can be a significant driver of viral evolution and host–pathogen interactions in honeybees.

## 1. Introduction

Antagonistic relationships between disease-causing agents, such as pathogens and parasites, and their hosts are driven by complex interactions modulated by ecological and evolutionary processes [1,2]. Both biotic and abiotic factors can influence disease outcomes and impose selective pressures on both host and pathogen, shaping coevolutionary dynamics across different contexts [3]. Understanding how these reciprocal exchanges interplay at the genome level is critical for combating epidemics, supporting agricultural systems and protecting vulnerable species in a changing global climate [4,5].

Population declines in insects broadly, and, particularly, in some insect pollinator species have been increasingly documented in recent decades [6,7]. One such pollinator species, the Western honeybee (*Apis mellifera*), while not demonstrating overall population declines, has observed a marked increase in colony mortality in recent years [8]. Research into honeybee colony mortality has identified multiple factors linked to declining bee health [9]. Some factors, as well as their synergistic interactions, include human-driven landscape changes (which reduce the availability and diversity of the flowering plants bees depend on for food) [10], chronic low-level pesticide exposure [11], climate change [12] and disease [13–15]. The dual epidemics of *Varroa destructor* mites and deformed wing virus (DWV) are the primary stressors driving global honeybee colony mortality, particularly in temperate regions of the USA and Europe [16]. *Varroa destructor*, an ectoparasite that reproduces on developing bee pupae, expanded its host species from just the Eastern honeybee, *Apis cerana*, to also the Western honeybee, *A. mellifera*, in the last century [14]. The introduction of *Varroa* to *A. mellifera* not only introduced a novel parasite with no coevolved resistance, but also introduced a novel transmission route to a historically benign, but now virulent, global pathogen: DWV [17]. Both DWV and *Varroa* have successfully spread to honeybee populations around the world [18–20], synergistically undermining honeybee health at multiple levels [21,22].

*Varroa*-mediated DWV transmission leads to increased titres, resulting in enhanced viral disease [21,23,24]. High levels of DWV lead to deformed wings in adults, reduced activity and ability to contribute to colony tasks and increased adult mortality [25–27]. This increased mortality leads to reduced colony survival, particularly in the winter months [28–30]. DWV strain diversity and evolution add further complexity to this system [31–33]; its two main strains, *deformed wing virus A* (DWV-A) and *deformed wing virus B* (DWV-B) as well as their recombinants [23,34], can differ in relative virulence [35–37], their molecular dynamics [38,39], ability to replicate in the *Varroa* vector [40] and epidemiology, with DWV-B displacing the previously dominant DWV-A across the globe [41].

Without management interventions to reduce levels of *Varroa*, most colonies succumb to mite infestations and associated viral infections within 2–3 years [19,42,43]. Indeed, wild, unmanaged honeybee colonies were decimated when *Varroa* was introduced to the USA and Europe in the past decades [44,45]. Recently, though, distinct honeybee populations across the globe have been found to self-sustain and persist despite ubiquitous stressor exposure [14,46]. One such mite-surviving population is located within the Arnot Forest outside Ithaca, NY, USA. Historically, these isolated, wild colonies located within the Arnot Forest have been found to be genetically distinct from bees from nearby apiaries [47]. While these bees persist without management, they do not demonstrate slowed or reduced mite reproduction [48] common to other mite-surviving populations [49]. Studies have suggested that these wild colonies are smaller in size than managed honeybee colonies, and more likely to swarm (a process of colony reproduction by fission which temporarily ceases brood production): both traits are associated with less brood in the colony and therefore fewer opportunities for mites to reproduce [50]. Swarming behaviour was not previously found to be consistently associated with decreased mites in honeybee colonies on the Island of Gotland, Sweden [51]. Therefore, these traits may not be the only factors that support the survival of wild honeybee colonies in the presence of *Varroa* infestation.

How is it possible for these feral bee populations to survive despite the presence of *Varroa* and DWV? While there is evidence for selection on the genome of the Arnot bee populations [52], it does not seem to have resulted in significant physiological resistance to mites [48]. It is possible that, rather than selection on the honeybee or the parasitic *Varroa* mite, pathogens including viruses have undergone rapid change to produce an avirulent infection phenotype, allowing for persistence of both host and disease-causing agents. Both mite-resistant populations on the Island of Gotland, Sweden, as well as unmanaged feral bees in Pennsylvania, USA, have been shown to survive high DWV infection levels [53,54]. In the Gotland bee populations, DWV-B is rarely detected compared to DWV-A [54,55]. This survival, therefore, could be due to virus-tolerant bee genotypes and/or adaptively avirulent virus populations.

It is predicted that in populations where a virus cannot readily infect new hosts, i.e. where population size is small or hosts (i.e. colonies) are far apart, highly virulent pathogens would be selected against, since infected hosts may succumb to the virulent disease prior to transmission to the next host [56–59]. Thus, less virulent viruses are expected to have a selective advantage and persist because their hosts would survive long enough to allow transmission [20,60]. While lower colony density in managed apiaries is not predicted to dramatically reduce disease prevalence, due to predicted high transmissibility (i.e. basic reproduction number, R0) of pathogens such as *Nosema* spp. and DWV [61], these conditions may be met in the Arnot Forest, as colonies here are smaller, more spread out and more apt to swarm than colonies in most managed apiaries [47,48,50]. Additionally, if pathogen spread among wild colonies is primarily by vertical transmission [17], at the individual level (i.e. queen to egg), group level (i.e. from parent colony to daughter colony) or after mating with infected drones, then this might also select for decreased virulence [62,63]. Thus, the viral populations circulating within these small, low-density wild populations may have been selected for reduced virulence, allowing them to persist despite lower rates of transmission. Note, however, that increased horizontal transmission (i.e. among unrelated colonies) is predicted to select for increased virulence. Horizontal transmission can occur when bees, *Varroa* and/or virus-contaminated materials (such as food stores) are moved between colonies by beekeepers or by bees through robbing behaviours, or possibly when bees from different colonies forage together and share viruses on flowers, as long as hosts are healthy enough to forage [14,64].

In this study, we investigated whether there is evidence of decreased virulence of viruses found in a population of dispersed, wild colonies compared to populations of crowded, managed colonies. We first assessed the presence and titre of major honeybee viruses in bees sampled from the Arnot Forest, from managed apiaries in adjacent regions in New York, and from apiaries in nearby Pennsylvania. These viruses included DWV, the primary virus transmitted by *Varroa*, as well as black queen cell virus (BQCV), a common bee virus not associated with *Varroa* transmission [17]. From a subset of infected bees, we sequenced DWV genomes and assessed nucleotide differences across these populations to determine if virus isolates were distinct across groups at the nucleotide level. Furthermore, we assessed virulence differences of these DWV isolates in developing honeybees by conducting experimental infections and then measuring pupal and adult mortality as well as other infection phenotypes. Overall, this multi-level analysis of DWV provides initial evidence that selection for decreased DWV virulence may play a role in allowing isolated bee populations to persist despite being parasitized by *Varroa*.

## 2. Methods

### (a) Experimental design

The experiments presented in this study can be split into two parts. Part 1 examines the prevalence and sequence identity of viruses naturally infecting bees sampled from the Arnot Forest, New York, and from managed apiaries in New York and Pennsylvania. In Part 2, virus inocula isolated from the naturally infected bees from Part 1 are used for experimental infections in developing honeybee pupae to assess potential infection differences across isolates. Taken together, we are able to assess potential differences in incidence, genotype and virulence across viral isolates, focusing on DWV isolated from bees from managed colonies versus the historically isolated Arnot Forest bees.

### (b) Honeybee collections

**Figure 1.**
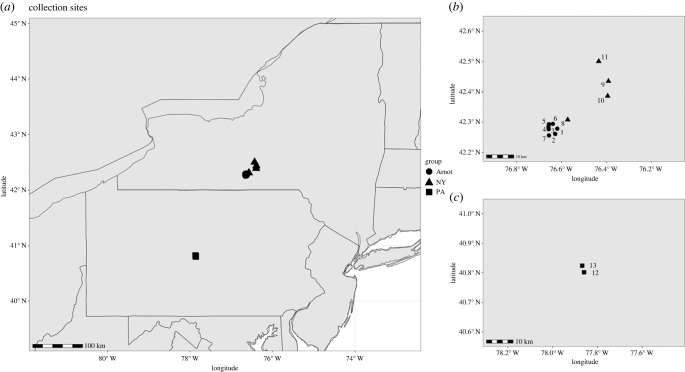
Sampling locations of bees assessed for native DWV infection. (*a*) The total sites across New York (NY) and Pennsylvania (PA), with a closer view of sites in NY (*b*) and PA (*c*). Within each group (distinguished by their point shapes), multiple sites were sampled. For both managed groups (NY and PA), the sites were apiaries from which multiple colonies were sampled.

Bees were collected from 13 sites across three different groups (based on location and management): Arnot Forest (Arnot), nearby New York Managed (NY) and Pennsylvania Managed (PA) (figure 1; electronic supplementary material, table S1). The Arnot collection sites were greater than 5 km from the closest NY managed site, but some less than 0.5 km from the forest edge. Honeybees often forage within 2.5 km but can forage up to 5 km away from their colony [65]. No information regarding other managed colonies that may have been kept within 5 km of the forest edge, representing potential viral transmission opportunities between managed and unmanaged Arnot forest bees, is currently available. Collections were conducted between September and 14 October 2019, between 10.00 and 17.00 on sunny, warm (approx. 18–24°C) days. Bees were captured using insect nets and kept on dry ice in conical tubes. Bees from managed colonies were collected from hive entrances, preferentially selecting obvious foragers, indicated by pollen-filled corbiculae. As it is technically challenging to locate wild colonies and collect foragers at the entrances of their nests, the Arnot Forest bees were collected while they were foraging on flowers. Upon returning from the field, bees were placed at −80°C for long-term storage.

### (c) Virus isolation

Viruses were isolated from individual bees as in [24]. Briefly, bees were homogenized in 500 µl of 1× PBS using a Bead Ruptor Elite (Omni International, Kennesaw, GA, USA) at 6.5 m s^−1^ for 45 s, then centrifuged for 3 min at maximum speed (greater than 15 000×*g*). The supernatant was passed through a sterile 0.2 µm syringe filter to separate viral particles from bee cells, and then was stored at −80°C until RNA purification.

### (d) Virus quantification by quantitative PCR

RNA was extracted from 30 µl of each virus inoculum using a Direct-zol RNA Miniprep kit (Zymo Research, Irvine, CA, USA) following the manufacturer's protocol. cDNA was prepared from 200 ng of RNA from each sample using a random primer method via the High-Capacity cDNA Reverse Transcription Kit with RNase Inhibitor (ThermoFisher Scientific, Waltham, MA, USA) following the manufacturer's protocol. cDNA was diluted 1 : 20× prior to quantitative PCR (qPCR) reactions. qPCR was conducted using PowerUp SYBR Green Master Mix (ThermoFisher) as in [24]. Virus was considered ‘present’ in an individual sample if the normalized mean Ct was less than 30. Primers can be found in electronic supplementary material, table S2, and data found in electronic supplementary material, tables S3–S9.

### (e) Sequencing and analysis of a subset of isolates

As BQCV was in low abundance across our samples, we focused on DWV for deep sequencing analysis. RNA extracts from a subset of virus isolations with higher DWV levels were submitted to the Pennsylvania State Genomics Core Facility (University Park, PA, USA) for library preparation and sequencing. Libraries were prepared from 28 samples and sequenced on the Illumina Miseq platform, resulting in 150 nucleotide paired-end stranded mRNA reads. Total reads per sample ranged between 274 942 and 753 035. Reads were assessed for quality with FastQC (v.0.11.9) and quality trimmed with Trimmomatic (v.0.39, option SLIDINGWINDOW:4 : 25) (electronic supplementary material, table S10).

Consensus DWV-A and DWV-B genomes were built using methods described in Ray *et al*. [24]. Briefly, genomes were created by aligning reads from each sample to DWV-A and -B reference genomes from NCBI (Ref. NC_004830.2 and NC_006494.1) using Hisat2 (v.2.1.0) [66]. Using bcftools (v.1.8) [67], variants were called and the consensus fastq sequence files were generated, and from the resulting fasta files, bases with qualities less than 20 were converted to Ns using seqtk (v.1.3-r106) [68]. DWV levels were low in these samples (electronic supplementary material, table S11), but full-length genomes could be constructed for 10 samples. This resulted in 11 consensus genomes (one sample, 13-1-E, was naturally co-infected with DWV-A and DWV-B). Reads were also aligned to a third variant of DWV, variant C, as well as other common bee viruses. However, less than 0.06% of reads within each sample aligned to DWV-C (CEND01000001.1), which could also be due to natural variation in DWV-A and -B or alignment errors. Fewer than 0.35% of reads within each sample aligned to other common bee viruses in the USA (acute bee paralysis virus (NC_002548.1), BQCV (NC_003784.1), chronic bee paralysis virus (NC_010711.1), Israeli acute paralysis virus (NC_009025.1), Lake Sinai virus 2 (NC_035467.1), sacbrood virus (NC_002066.1)). These viruses were not further examined within the sequence data.

For phylogenetic analyses, multisequence alignments of consensus genomes and reference genomes (DWV-A reference (NC_004830.2), DWV-B reference (NC_006494.1) and DWV-C reference (CEND01000001.1)) were generated with Clustal Omega using default settings (v.1.2.3). As the DWV-B genome from isolate A-3 could not be assembled from the original sequencing, the DWV-B genome constructed from the propagated A-3 isolate from 2021 (see Experimental infection samples and procedure) was included in its place in the alignment but not in the variant calling analysis. Multisequence alignment was then imported into MEGAX (v.10.1.8) for maximum-likelihood tree construction using the Tamura–Nei substitution model at the nucleotide level, and bootstrapped using 1000 replicates [69]. Consensus genomes, as well as raw sequence reads, can be found on the NCBI Genome and SRA database (PRJNA922567 and PRJNA922218, genome accessions OR497372–OR497398). Variants within DWV-A and -B populations were called using bcftools and annotated using SNPeff (v.5.0) [70] as in Ray *et al*. [24] (electronic supplementary material, tables S12–S14).

### (f) Experimental infection samples and procedure

Experimental infections were conducted from August to September 2021. Two different colonies (thus representing distinct genotypes) from a Penn State University research apiary were used. Prior to infection studies, colonies were assessed for viral infection via qPCR (electronic supplementary material, figure S4 and table S26); there was no or very low indication of common bee viruses. Both colonies were inspected weekly to confirm health status (i.e. no obvious signs of viral disease, and a low parasite load) and to confirm the presence of the original queen.

The DWV isolates used in experimental infections can be found in electronic supplementary material, table S15. To reach a sufficiently high titre of viral genotypes to conduct these experiments, inoculums were propagated in pupae collected from a DWV-free (assessed via qPCR) colony [71]. Pupae at the white-eyed stage (14 days post egg laying) were injected with the viral isolates, then collected on dry ice at 4 days post-injection (4DPI). Virus was isolated from individual pupae [24], and aliquoted to minimize the number of freeze–thaws. Isolates were assessed for DWV quantity and co-infection of BQCV and SBV. RNA from isolates was submitted for RNA sequencing to confirm minimal DWV sequence variation after virus propagation (electronic supplementary material, figure S2). Propagated virus inocula were normalized to two doses: approximately 5 × 10^6^ genome equivalents per μl (high) and approximately 5 × 10^2^ genome equivalents per μl (low). Virus being actively used was kept at 4°C for no longer than 3 days.

Pupae at the white-eyed stage were used for experimental infections. Any pupae that showed eye pigmentation (indicating older than 14 days old), melanization (indicating injury during collection) or *Varroa* within their cell were not used. Infections were conducted in a UV sterilized hood to minimize contamination by mould and other opportunistic microbes. One microlitre of the propagated virus inoculum was injected using a mouth aspirator with an attached 10 µl capillary tube pulled into a needle. Needles were changed between inocula to avoid contamination. To measure colony DWV levels and the effect of the injection itself on DWV levels, control bees (‘Control’, collected from the colony but not manipulated further) and PBS-injected bees (‘PBS-inject’, injected with the saline solution used for the stock viral isolation) were included as controls.

Injected pupae were kept in 48-well plates that were placed in a cabinet at 75% RH within an incubator at 34.5°C (Thermo Science Nalgene Acrylic Desiccator Cabinet). Subsets of samples were collected at 3 days post-injection (3DPI) to assess viral titres via qPCR. Pupae were monitored daily for mortality, and when nearing the time of eclosion (approx. 7DPI) they were monitored every 8–12 h for successfully eclosed bees. ‘Successfully’ pupated (also known as ‘eclosed’) bees surviving to adulthood were identified as ones having high mobility, i.e. notable movements around their respective wells (electronic supplementary material, tables S16 and S17). Once eclosed, bees were removed from their individual well with sterilized forceps, inspected for deformed wings (electronic supplementary material, table S18), and placed into Plexiglas cages (10 × 10 × 7 cm^3^), split by group (1–7 bees per cage, depending on eclosion rate), noting the time of transfer. Cages were provided 30% sugar water and honey, ad libitum, replenished daily as needed, and placed within an incubator at 34.5°C and approximately 40–60% RH. Cages with adult bees were monitored for survival daily, and bees that had perished were removed (electronic supplementary material, table S19).

### (g) Virus quantifications from experimental infections

RNA was isolated from abdomens from 3DPI pupae collected during the experimental infection experiments using the RNeasy Mini Kit (Qiagen, Hilden, Germany) following manufacturers' protocol including a DNAse incubation step and quantified using a Nanodrop. cDNA synthesis and qPCR were conducted as described above. The raw and processed data from the qPCR runs are contained in electronic supplementary material, tables S20–S26.

### (h) Statistical analyses

Statistical analyses were conducted in R (v.3.6.3) using the ‘stats’ package [72]. Pearson's *χ*^2^ test assessed frequency differences in viral presence across groups (Arnot, NY and PA) and one-way analysis of variance (ANOVA) compared viral loads of infected individuals across groups. Differences in viral loads in experimentally infected samples were assessed using two-way ANOVA across isolate and dose (electronic supplementary material, table S27). Pearson's *χ*^2^ test assessed differences in pupation survival rates and rates of deformed wings across isolates (electronic supplementary material, tables S28–S30). Kaplan–Meier survival analysis was conducted using the ‘survival’ (v.3.4.0) and ‘survminer’ (v.0.4.9) packages (electronic supplementary material, table S31).

## 3. Results

### (a) Deformed wing virus presence and loads do not differ between Arnot Forest and managed bee populations

**Figure 2.**
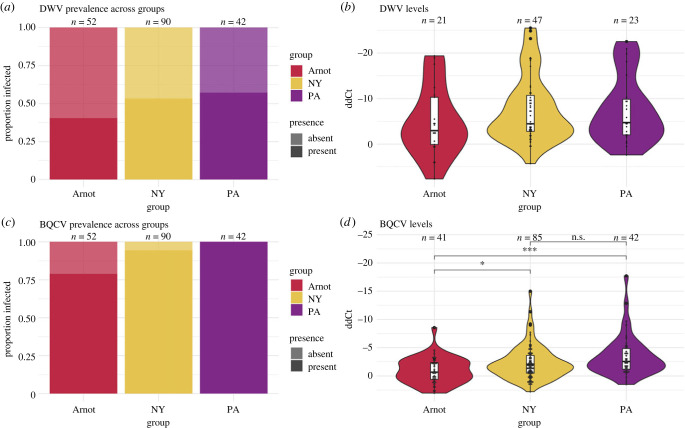
Levels of DWV and BQCV infection across managed bees and Arnot Forest bees. (*a*) The proportions of DWV-infected bees did not differ between the bees collected from the Arnot Forest versus from managed colonies in NY and PA. (*b*) Viral loads of DWV-infected bees were similar across groups. (*c*) Rates of BQCV were significantly lower in bees collected from the Arnot Forest. (*d*) Of infected samples, BQCV titres within individuals from the Arnot Forest were significantly lower than titres from bees collected from managed colonies. Bees were categorized as infected when normalized qPCR dCt was less than 30. The *Y*-axis is reversed in (*b*) and (*d*), as lower ddCt values are indicative of a higher starting template in qPCR reactions.

Viruses were detected and quantified from individual bees collected from the Arnot Forest and managed colonies in New York (NY) and Pennsylvania (PA). All groups had detectable levels of DWV and BQCV. Across groups, there was no significant difference in the prevalence of DWV, with approximately 40–57% infection rate (Pearson's *χ*^2^ test, *p*-value = 0.2061; figure 2*a*). When comparing viral loads between infected individuals, there was no significant difference in the infection level across groups, with all groups having a range of lowly and highly infected bees (one-way ANOVA, *p*-value = 0.353; figure 2*b*). Both master variants (i.e. strains) DWV-A and DWV-B were found across groups (electronic supplementary material, figure S1).

Compared to DWV prevalence, however, the incidence of BQCV was lower in the bees caught in the Arnot Forest (Pearson's *χ*^2^ test, BQCV *p*-value = 0.001; figure 2*c*), as were their viral titres (one-way ANOVA, BQCV *p*-value < 0.001), relative to the bees collected from managed colonies in NY and PA (figure 2*d*).

### (b) Evaluation of deformed wing virus genetic diversity across isolates

**Figure 3.**
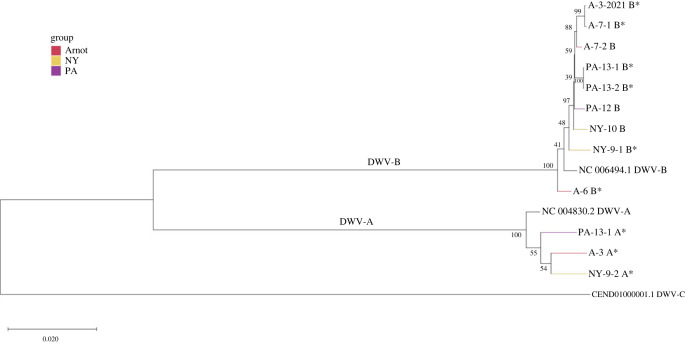
Phylogeny of DWV-A and DWV-B from bees collected in the Arnot Forest and from managed colonies in NY and PA. Maximum-likelihood trees with 1000 bootstrap replicates were generated from each isolate's consensus genome along with reference genomes for DWV-A, DWV-B and DWV-C (NC_004830.2, NC_006494.1 and CEND01000001.1). Nodes are coloured by group. Stars indicate isolates used in experimental infections. As the DWV-B genome from isolate A-3 could not be assembled from the original sequencing, the propagated DWV-B from 2021 is shown instead.

Isolates with high levels of detectable DWV were subjected to RNA sequencing to identify nucleotide variation across viral genomes. Of the 28 sequenced samples, 10 had sufficient numbers of reads aligning to DWV to allow for the reconstruction of full viral genomes, resulting in 3 DWV-A sequences and 8 DWV-B sequences. These consensus genomes clustered by master variant identity (i.e. DWV-A and DWV-B) in phylogenetic analyses of whole genomes (figure 3). In the two instances where we had multiple DWV-B isolates collected from the same site (Site 7: A-7-1 and −2, Site 13: PA-13-1 and −2), the consensus genomes isolated from the same site also clustered together; otherwise, there is no obvious clustering at the level of geographical location or by group (figure 3).

**Figure 4.**
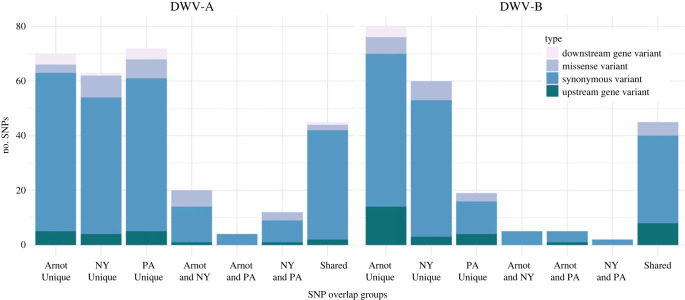
Comparison of single nucleotide polymorphisms (SNPs) identified in DWV isolates. SNPs compared to the DWV-A (left) or DWV-B (right) reference genomes were identified within individual isolates. Lists of SNPs found within each group (Arnot, NY and PA) were then compared across groups to determine which were unique to each group (e.g. ‘Arnot Unique’), shared between two groups (e.g. ‘Arnot and NY’), or found across all groups (‘Shared’). Type of variant (upstream or downstream of the DWV polyprotein coding region, or missense or synonymous variants within the coding region) is indicated by colour.

Isolates represented primarily by DWV-A show approximately 1.3–1.4% variation across the genome compared to the DWV-A reference genome (NC_004830.2). All isolates had some single nucleotide polymorphisms (SNPs) that were unique to each isolate compared to the reference genome, as well as some variation from the reference that were shared across groups (figure 4; electronic supplementary material, table S14). DWV-B isolates had about 0.7–0.8% variation compared to the DWV-B reference (NC_006494.1); DWV-B isolates also contained SNPs unique to each isolate, as well as shared within and across groups (figure 4; electronic supplementary material, table S14).

Notably, there is one unique, non-redundant variant identified in all three Arnot Forest DWV-B isolates in this analysis: Val896Ile, resulting in a conservative amino acid change in the putative capsid protein region of the DWV genome (electronic supplementary material, figure S3). This variant was not identified in any of the isolates from managed bees in this analysis. Overall, SNPs were identified across all groups across the genome, and any missense mutations in DWV-A and DWV-B isolates tended to represent conservative amino acid changes with respect to the reference genome, including the Val896Ile variant found in the Arnot isolates (electronic supplementary material, tables S12–S14).

### (c) Infection phenotypes varied across deformed wing virus isolates and between doses

Seven DWV isolates, representing a DWV-A and DWV-B from each group, were further assessed for phenotypic differences through experimental infections: three isolates from Arnot Forest samples, two isolates from NY (from Colony 2 at Site 9) and two isolates from PA (from Colony 1 at Site 13). White-eyed pupae were injected with high doses (approx. 5 × 10^6^ genome equivalents per μl) or low doses (approx. 5 × 10^2^ genome equivalents per μl) of an isolate, or 1× PBS, as sham-injection controls (PBS) and uninjected pupae as full controls (Control). At 3DPI, subsets of pupae were collected to assess infection titres. The remaining bees were allowed to further develop, and assessed for infection phenotypes including: eclosion rates, rates of symptomatic deformed wings and adult survival through time.

Viral loads at 3DPI varied across DWV+ groups (two-way ANOVA, *p*-value < 0.001) and were generally higher than Controls (electronic supplementary material, figure S4 and table S27). DWV load was not significantly different across dose or colony (two-way ANOVA, *p*-value = 0.0716 and 0.234, respectively). Of these seven isolates, three were found contaminated with other bee-infecting viruses: A-7-1 and NY-9-1 with high levels of BQCV, and NY-9-2 with highly detectable paralysis virus (electronic supplementary material, table S26).

**Figure 5.**
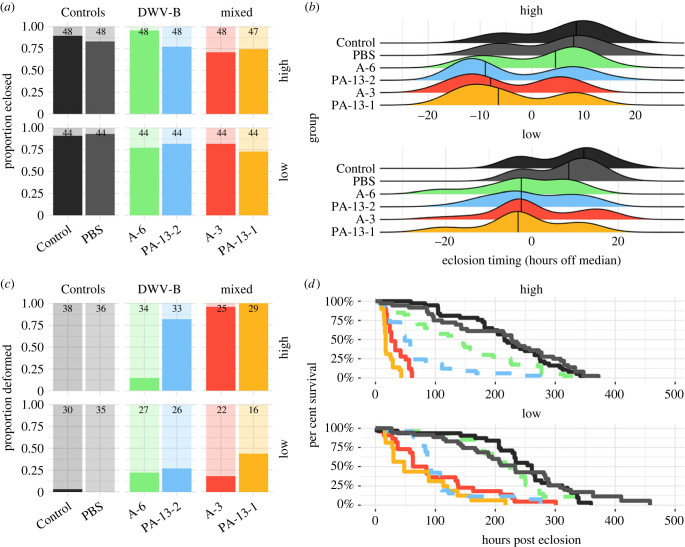
Disease symptoms varied across DWV isolates and dose. (*a*) Eclosion percentages were high and similar across groups. DWV-infected bees tended to eclose faster than controls (*b*). Percentages of deformed wing bees varied across isolates (*c*) and survival percentages were generally higher (*d*) in bees exposed to Arnot Forest isolates relative to bees exposed to managed colony isolates. Samples sizes for eclosion (*a*) and deformed wing rates (*c*) can be found above each bar.

Eclosion rates, i.e. pupal survival, were similarly high (between 71% and 96%) across all groups and doses (figure 5*a*; electronic supplementary material, table S28), except for the three groups that had other contaminating viruses, where pupation rates were low (between 0% and 12%) (electronic supplementary material, table S16); due to low survival to adulthood, these three contaminated groups were subsequently removed from further symptom screening. This resulted in a pairwise-comparison of infection dynamics for 4 DWV+ isolates: Arnot versus PA managed DWV-B (A-6 versus PA-13-2) and Arnot versus PA managed mixed (i.e. DWV-A/DWV-B) (A-3 versus PA-13-1).

Interestingly, we saw more rapid pupation rates across our 4 DWV+ groups compared to Controls (figure 5*b*; electronic supplementary material, table S29), as has been reported previously [73].

Of bees that successfully pupated, those in the 4 DWV+ groups had higher rates of symptomatic deformed wings compared to those that were in the Control group (figure 5*c*; electronic supplementary material, table S30). Generally, mixed groups had higher deformed wing rates compared to DWV-B isolates (figure 5*c*).

Adult survival over time showed the most distinct disease phenotypes across DWV+ groups (figure 5*d*; electronic supplementary material, figure S5). In the high dose experiments, all the DWV+ groups had significantly lower survival than Controls (PBS and Control), but the Arnot isolate samples consistently had better survival than their managed isolate counterparts. In the low dose, samples infected with isolate A-6 performed similarly to Controls, while the other DWV+ groups again had significantly worse survival than Controls, but were not significantly different from one another (electronic supplementary material, table S31).

## 4. Discussion

We investigated whether adaptive decreased virulence of DWV may contribute to the ability of the feral honeybees of the Arnot Forest to survive. No significant differences were found between Arnot Forest and managed bees in either their DWV infection rates or their viral loads. However, sequence analyses of DWV isolates revealed unique SNPs associated with the viruses in the different groups of bees. Furthermore, in experimental infections, we found differences—across multiple metrics of virulence—among bees infected with different DWV isolates. Most notably, we found that infections with DWV isolates collected from Arnot Forest bees generally resulted in milder symptoms and better survival compared to infections with DWV isolates collected from managed colonies. Overall, this study provides initial evidence of relatively low virulence of DWV circulating within the Arnot Forest. This is a potential mechanism for colony survival in this forest, despite *Varroa* infestations and pathogen pressure.

By examining individuals, we were able to measure fine-scale infection rates across all groups, and by examining DWV-infected foragers, we began to evaluate which viral genotypes may be circulating in the Arnot Forest. All three groups had detectable DWV and BQCV, which shows that the survival of the Arnot Forest bees is not due to a lack of pathogen pressure. Lower levels of DWV infection might explain the ability of Arnot Forest bees to persist without management, but we found about the same DWV infection rate (approx. 50%) in foragers across all three groups. An ability to suppress DWV titres might also be an adaptation associated with survival, but we found no evidence of this, as viral loads in infected individuals were similar across all three groups, consistent with other studies comparing the viral loads of workers in feral versus managed colonies [53,74,75]. Our results suggest that the Arnot Forest bees are instead able to tolerate high levels of infection, as do other bees with mite-resistant genotypes that have demonstrated DWV tolerance [54,76–78].

The infection rates and titres of BQCV were lower in Arnot Forest bees versus managed bees, though the rates we found are still high (78.8% infected; figure 2*c*). BQCV is not associated with vector transmission by *Varroa* [27]. BQCV is commonly found in honeybee colonies across the globe [27,79,80], and usually is not associated with high worker mortality [81]. Therefore, the relatively low infection rates of BQCV may contribute somewhat to Arnot Forest bee survival, but probably it is not the primary basis for bees' survival. BQCV is readily transferred between bees foraging together in a patch of flowers [82], so it is not surprising that it is found in wild colonies. BQCV rates become high where there are high densities of honeybee colonies [83,84]. Thus, the lower levels of BQCV in the Arnot Forest bees may reflect reduced horizontal transmission between foragers from managed and wild colonies, perhaps due to relatively low densities of honeybee colonies within the Arnot Forest [47,48,50].

The Arnot sites were greater than 5 km from the closest NY managed site, but some less than 0.5 km from the forest edge. While honeybees can forage up to 5 km from their hive, most bees forage less than 2.5 km from their colony [65]. Thus, if there were managed bees kept near the forest edge, there could have been an overlap of foragers between managed and unmanaged colonies on these flowers. This could explain why DWV infection rates were similar across groups, both due to viral transmission between managed and unmanaged bees during co-foraging, as well as capturing both managed and unmanaged foragers during our collections. Though the BQCV rates in our presumed Arnot Forest bees were lower compared to the nearby NY apiaries, indicative of a separate bee population, this may be an effect of only the healthiest bees (also known as bees with the lowest BQCV loads) being able to reach the resources within the Forest. As there are no available maps of total managed apiaries in that area, though, we cannot confirm this transmission potential. Standardized collections at Arnot Forest colony entrances, although technically challenging, can help to better confirm the colony identity of all bees in future studies.

Both DWV-A and DWV-B genotypes were identified across all three groups. Both master variants, and their recombinants, are virulent [23,39,85], which shows that the survival of the Arnot Forest bees is not due to the absence of a particular master variant. At the genome level, consensus genomes did have unique variation across the isolates from the three different groups of colonies, which indicates that there are indeed distinct DWV genotypes circulating in the Arnot Forest. However, consensus DWV-B genomes from the Arnot Forest did not fully cluster with one another, so there does not appear to be an ‘Arnot Forest’ sequence variant at the whole-genome level. Similarly, the DWV-A populations in the isolated, mite-resistant colonies in Sweden cluster in the 2009–2010 samples, but not in the 2015 sample [55,86]. Additional sampling over multiple years may reveal more consistent patterns of DWV genotypes within the Arnot Forest.

A small portion of identified SNPs is shared across all isolates within their group, including a predicted missense variant in the capsid region of the Arnot Forest DWV-B genomes. Mutations in DWV capsid proteins may affect virus cell entry or recognition by the host [87]. Given that this predicted variant results in an amino acid change within the same functional group (valine to isoleucine), it is unclear what effect this SNP may have, if any. Many of the missense variants identified in these populations do not appear to produce a functional change, as no SNPs were identified in putative functional regions and most amino acid substitutions are still within similar functional groups. Nonetheless, sub-consensus and synonymous variation can play important roles in translational efficiency (e.g. codon bias) [88], RNA secondary structure [89] and pathogen fitness and adaptability [90], and these may influence the viral dynamics of the Arnot Forest isolates.

While this study presents the first evidence of individual variation in virulence within DWV master variants, we were limited to only assessing four viral isolates through adulthood: two Arnot Forest isolates and two PA managed isolates. We screened 184 individual bees with qPCR and identified 28 bees with relatively high levels of DWV; of these samples, only 10 bees had sufficiently high levels to allow for viral genome assembly. Of these, seven isolates were propagated for experimental infections. Only four of these isolates did not have other viruses and thus could be used for analysis. Thus, in future studies, a much larger sample set of bees should be collected and evaluated, to ensure a larger number of isolates representing different populations.

Our results provide initial evidence of less virulent DWV populations within bees of the Arnot Forest, or alternatively, more virulent DWV populations in PA managed bees. The isolate with the most distinct infection outcomes, A-6, was also the most diverged DWV-B in the phylogenetic analysis. It is not clear how infection would compare with more genetically similar genotypes, as the other DWV-B isolates we assessed were co-infected, resulting in worsened disease. Moreover, since the A-6 genotype is highly distinct from both its counterpart in the experimental infections as well as other Arnot DWV-B genotypes, it may, therefore, not be representative of the sum of Arnot Forest viral population dynamics, *per se*, and may represent instead a unique variant within the DWV-B classification. To explore adaptive viral avirulence as a mechanism whereby honeybee colonies survive *Varroa* infestations, additional DWV genotypes, from both within and beyond the Arnot Forest, need to be assessed. This could help us to better understand phenotypic variation in infection effectiveness within and across DWV master variants.

The experimental infections reported here also provide guidance for future studies in DWV virulence. Overall, the pupation survival rates were comparable for DWV-infected bees and control bees, which has been observed in other studies [35,91]. However, the rates of deformed wings and adult survival through time differed among DWV+ groups, indicating the importance of measuring a panel of symptoms during disease phenotyping. We did not find an Arnot Forest isolate that was fully avirulent, although exposure to a low dose of the Arnot Forest isolate A-6 resulted in adult bee survival that nearly matched that of controls. Samples infected with co-infection isolates performed worse than samples infected with DWV-B alone. BQCV has also been shown to be highly virulent when injected directly into the haemolymph of worker bees [92,93]. Furthermore, co-infection of DWV variants can result in increased adult mortality through time, which is consistent with our previous observations of highly virulent DWV-A + DWV-B populations [24], and may explain the low rates of DWV co-infection in the naturally infected individuals across all groups (electronic supplementary material, figure S1).

It is important to note that our study tested the impacts of infection with different DWV isolates on honeybees derived from managed stocks. It is possible that the Arnot Forest bees and DWV have co-evolved to be adapted to one another [94], so there might be even lower virulence in experimental infections of Arnot Forest bees with Arnot DWV isolates. Indeed, honeybee host genotype has been an important factor in DWV infection studies [73,78,95], and our study further uncovers how DWV genotype, even within master variant groups, can differ in infection severity. Future studies examining local adaptation [96] and genotype-by-genotype interactions [97,98] will reveal fundamental characteristics of host–pathogen dynamics and avenues for supporting honeybee health.

The relationship among honeybees, *Varroa* mites, viruses and beekeepers provides a fascinating system in which to study host–pathogen dynamics and evolution [59,64]. The introduction of *Varroa* mites provided a novel mechanism for horizontal viral transmission which accelerated the spread of DWV, both within and between colonies, and especially in managed operations [14,18]. There has been considerable focus and interest in selecting for honeybee genotypes that are resistant to both *Varroa* and DWV [14]. However, within populations of wild honeybee colonies, decreased opportunities for both horizontal and vertical transmission may result in selection for less virulent viral genotypes [99], which may be a novel approach to supporting honeybee health. Our study provides the first evidence for this mechanism, and lays the groundwork for further studies examining these dynamics in populations of both managed and wild colonies, and for potentially identifying biomarkers of less virulent DWV populations.

## Data Availability

Data are available from the Dryad Digital Repository: https://doi.org/10.5061/dryad.8pk0p2ntr [100]. Raw sequence reads (NCBI): PRJNA922567 and PRJNA922218. Viral genome accessions (NCBI): OR497372–OR497398. A previous version of this paper is available on *bioRxiv* [101]. Supplementary material is available online [102].
